# Multiple Molecular and Cellular Mechanisms of Action of Lycopene in Cancer Inhibition

**DOI:** 10.1155/2013/705121

**Published:** 2013-07-21

**Authors:** Cristina Trejo-Solís, Jose Pedraza-Chaverrí, Mónica Torres-Ramos, Dolores Jiménez-Farfán, Arturo Cruz Salgado, Norma Serrano-García, Laura Osorio-Rico, Julio Sotelo

**Affiliations:** ^1^Departamentos de Neuroinmunología, Instituto Nacional de Neurología y Neurocirugía (INNN), C.P. 14269, Mexico City, DF, Mexico; ^2^Neurobiología Molecular y Celular INNN-UNAM, Instituto Nacional de Neurología y Neurocirugía (INNN), C.P. 14269, Mexico City, DF, Mexico; ^3^Facultad de Química, Universidad Nacional Autónoma de México (UNAM), C.P. 04510, Mexico City, DF, Mexico; ^4^Unidad Periferica de NeuroCiencias INNN-UNAM, Instituto Nacional de Neurología y Neurocirugía (INNN), C.P. 14269, Mexico City, DF, Mexico; ^5^Facultad de Odontología, Universidad Nacional Autónoma de México (UNAM), C.P. 04510, Mexico City, DF, Mexico; ^6^Neuroquimica, Instituto Nacional de Neurología y Neurocirugía (INNN), C.P. 14269, Mexico City, DF, Mexico

## Abstract

Epidemiological studies suggest that including fruits, vegetables, and whole grains in regular dietary intake might prevent and reverse cellular carcinogenesis, reducing the incidence of primary tumours. Bioactive components present in food can simultaneously modulate more than one carcinogenic process, including cancer metabolism, hormonal balance, transcriptional activity, cell-cycle control, apoptosis, inflammation, angiogenesis and metastasis. Some studies have shown an inverse correlation between a diet rich in fruits, vegetables, and carotenoids and a low incidence of different types of cancer. Lycopene, the predominant carotenoid found in tomatoes, exhibits a high antioxidant capacity and has been shown to prevent cancer, as evidenced by clinical trials and studies in cell culture and animal models. *In vitro* studies have shown that lycopene treatment can selectively arrest cell growth and induce apoptosis in cancer cells without affecting normal cells. *In vivo* studies have revealed that lycopene treatment inhibits tumour growth in the liver, lung, prostate, breast, and colon. Clinical studies have shown that lycopene protects against prostate cancer. One of the main challenges in cancer prevention is the integration of new molecular findings into clinical practice. Thus, the identification of molecular biomarkers associated with lycopene levels is essential for improving our understanding of the mechanisms underlying its antineoplastic activity.

## 1. Introduction

Cancer is a disease that is initiated by a series of cumulative genetic and epigenetic changes that occur in a normal cell. Cancer development is characterised by three stages: initiation, promotion, and progression [1]. Initiation is defined as a rapid and irreversible process that begins with genotoxic DNA damage resulting from exposure to endogenous or exogenous carcinogens. The initiation of chemically induced tumourigenesis involves carcinogenic metabolic activation and the subsequent covalent modification of the genomic DNA, allowing the activation of oncogenes or the inactivation of tumour suppressor genes. Tumour promotion is a reversible process characterised by the transformation of an initiated preneoplastic cell due to an epigenetic alteration resulting from chronic exposure to tumour promoters (i.e., growth factors, hormones, and UV radiation). The final stage of the neoplastic transformation, progression, involves tumour growth with the potential for invasion and metastasis.

Four possible strategies have been described for the reduction of cancer incidence and cancer-related mortality: (I) prevention, (II) early diagnosis, (III) improvements on treatment, and (IV) improved detection of nondiagnosed cancers [2]. Of these options, prevention is the most favourable. Between 30 to 40% of cases are reportedly preventable through diet modification, adequate body weight management, and physical activity [3]. 

Chemoprevention aims to stop or to reduce cancer development by intervening in the carcinogenesis process via the administration of natural or synthetic agents [4]. Chemopreventive compounds have been classified into two groups: blocker and suppressor agents. The first group blocks cancer initiation, whereas the second stops or delays the promotion and progression of preneoplastic or malignant cells [5]. 

Chemopreventive compounds intended for human use must not elicit significant secondary effects. They must be efficient against multiple molecular targets, and their mechanism(s) of action should be well defined. They also should be inexpensive and accessible. In addition, chemopreventive compounds should ideally take the form of an oral agent appropriate for widespread application in the general population. These characteristics contrast with those of chemotherapeutic agents, which are usually very expensive and toxic and are generally applied when the disease has already disseminated, so they are less efficient [6].

Epidemiological studies have suggested that the inclusion of fruits, vegetables, and whole grains in dietary intake might prevent and even reverse the cellular changes associated with carcinogenesis at the initial stages, thus reducing tumour incidence [4]. These encouraging results have been documented *in vitro* and *in vivo*, as well as in clinical trials [7]. These beneficial data are attributed to bioactive compounds, including both essential nutrients (i.e., selenium, calcium, zinc, and vitamins C, D, and E) and nonessential components (carotenoids, flavonoids, allyl sulfide indole compounds, conjugated acids and n3 fatty compounds). Bioactive compounds have been reported to modify specific carcinogenic processes, including cancer metabolism, hormonal balance, transcription factors, cell-cycle control, apoptosis, inflammation, angiogenesis, and metastasis. Furthermore, natural compounds might modulate cellular processes by acting on molecular targets, such as AP-1 (activator protein-1), STATs (signal transducers and activator of transcription), NF-*κ*B (nuclear factor-kappa B), HIF (hypoxia inducible factor), Nrf2 (nuclear factor-erythroid 2- related factor 2), MAPKs (mitogen activated protein kinases), PI3K/AKT (phosphoinositide 3-kinase/AKT), and VEGF (vascular endothelial growth factor), thereby favouring the inhibition of carcinogenesis. Here, we review the antineoplastic effects and the mechanism of action of lycopene, a carotenoid that is abundant in fruits and vegetables.

## 2. Lycopene and Cancer 

Lycopene is a natural pigment synthesised by photosynthetic plants and microorganisms [8]. It is a highly unsaturated acyclic isomer of *β*-carotene; its hydrocarbon chain contains 11 conjugated and 2 nonconjugated bonds [9]. Lycopene is the most abundant carotenoid in tomatoes and is present in concentrations ranging from 0.9 to 9.27 mg/100 g depending on the variety [10, 11]. Tomato paste is a more concentrated source of lycopene (51–59.7 mg/100 g) than nonprocessed tomatoes [11]. Other sources of lycopene are found in red fruits, such as rosehips, watermelons, red grapefruits, papayas, apricots, and pink guavas [12]. 

Epidemiological studies support the possibility that lycopene can reduce cancer risk. In a meta-analysis of 72 studies, 57 reported an inverse association between lycopene ingestion and the risk of diverse cancer types (including prostate, breast, lung, and colon), and 35 studies reported statistically significant associations. None of these studies reported any adverse effects of increased tomato intake or increased circulating lycopene levels [13]. In another study, lycopene was found to be the only micronutrient present in the human serum associated with a decreased risk for breast cancer [14].

In a randomised clinical trial, lycopene was applied as a therapeutic agent for prostate cancer [15]. In that study, patients received 30 mg of lycopene daily over a period of three weeks prior to undergoing radical prostatectomy. Eighty percent of the patients supplemented with lycopene presented with smaller tumours compared with the control patients. Moreover, the plasma levels of the specific prostatic antigen decreased by 18% in the supplemented patients, whereas they increased by 14% in the control group. These results indicate that lycopene supplementation might represent an adjuvant treatment for this disease.

In addition to the correlation between lycopene and prostate cancer, increasing evidence suggests that lycopene plays an important role in cancer prevention in other organs, such as the breast, lung, gastrointestinal tract, pancreas, uterine cervix, and the ovaries [13]. Although the antioxidant properties of lycopene were thought to be primarily responsible for its biological effects, other mechanisms have also been identified. The following review is an abbreviated overview of *in vitro* and *in vivo* evidence supporting the contribution of lycopene to cancer prevention.

## 3. Potential Mechanisms of Actions of Lycopene

### 3.1. Antioxidant Activity

Oxidative stress is caused by the increased production of reactive oxygen (ROS) and nitrogen (RNS) species, including superoxide (O_2_^•−^), hydroxyl (OH^•^), peroxyl (ROO^•^), alkoxyl (RO^•^) and peroxynitrite (ONOO^•^), as well as nonradical species, such as singlet oxygen (^1^O_2_), ozone (O_3_), and hydrogen peroxide (H_2_O_2_) [16]. These chemical species are generated by a wide variety of processes, including mitochondrial respiration, ischemia/reperfusion, inflammation, and the metabolism of exogenous compounds [17]. The excessive generation of ROS might oxidise cellular biomolecules, including carbohydrates, proteins, lipids, and DNA. The oxidation of these molecules can facilitate carcinogenesis-related processes, such as cellular transformation, proliferation, apoptosis resistance, angiogenesis, and metastasis via genetic alterations, including DNA damage, mutation, epigenetic changes, and genetic instability [18].

Because they contain many double-conjugated bonds, natural carotenoids display strong antioxidant capacity [19]. Lycopene possesses robust antioxidant activity that is exerted via different mechanisms. For example, lycopene can trap ^1^O_2_. Moreover, lycopene has been shown to be twice as effective as *β*-carotene and 10 times more efficient than *α*-tocopherol in its ability to trap ^1^O_2_ [20]. Another mechanism that accounts for the antioxidant activity of lycopene is its reaction with free radicals [21]. Lycopene has also been suggested to act as an antioxidant *in vivo* by repairing radicals derived from vitamins E and C [22]. Matos et al. [23] reported that lycopene protects mammalian cells against lipid peroxidation and oxidative DNA damage induced *in vitro* by an iron chelant. In addition, *in vivo* studies have shown that mitochondrial DNA damage caused by ROS generation through UV radiation is partially blocked by tomato sauces rich in lycopene [24]. Muzandu et al. [25] reported that in Chinese hamster lung fibroblasts, lycopene can inhibit the DNA damage and protein nitration caused by peroxynitrite generated by 3-morpholinosydnonimine, suggesting that lycopene might quench or bind to either peroxynitrite or its intermediates. Liu et al. [26] showed that in lycopene-treated prostate cancer cells, lycopene localised predominantly within the nuclear membrane, consistent with its protective effect.

#### 3.1.1. Regulation of Antioxidant Response Element (ARE)

A previous study proposed that the effects of lycopene may be attributed to the induction of antioxidant enzymes and phase II detoxifying enzymes [27]. The administration of lycopene (2.5 mg/kg) can significantly suppress gastric cancer *in vivo* by reducing lipid peroxidation, increasing the levels of the antioxidants vitamin C, vitamin E, and reduced glutathione (GSH), and increasing the activity of circulating GSH-dependent enzymes, such as the glutathione peroxidase (GPx), glutathione reductase, and glutathione-S-transferase (GST) [28]. Lycopene also blocks experimental buccal carcinogenesis by inhibiting oxidative stress via the upregulation of detoxification pathways [29].

The transcriptional upregulation of the genes encoding the antioxidant and phase II detoxifying enzyme is mediated by cis-acting DNA sequences located within their promoter regions that are known as antioxidant response elements (AREs). The major ARE transcription factor is Nrf2 (nuclear factor E_2_-related factor 2), which plays an important role in the detoxification of carcinogenic agents and in the modulation of the antioxidant cellular defence system, as it promotes the upregulation of stress-induced cytoprotective enzymes including NAD(P)H:quinone oxidoreductase-1 (NQO1), superoxide dismutase (SOD), GST, GPx, heme oxygenase-1 (HO-1), glutamate cysteine ligase (GCL), catalase, and thioredoxin (Tx1). In addition, Nrf2 also elicits anti-inflammatory effects [27].

Under normal conditions, Nrf2 is localised in the cytoplasm, forming a complex with the inhibitory protein Keap1 (Kelch-like ECH-associated protein 1) [30]. However, oxidative stress promotes the dissociation of Nrf2 from Keap1, rescuing Nrf2 from proteasomal degradation and inducing its nuclear translocation, allowing it to bind to AREs together with other transcription factors to regulate the expression of the target genes [31].

In MCF-7 and HepG2 cells, lycopene increases the mRNA and protein levels of NQO1, GCL, and GSH by activating Nrf2 [27]. In addition, the ethanolic extracts of lycopene activate ARE-regulated reporter genes with a potency similar to that observed for pure lycopene. These findings suggest that oxidised metabolites might be responsible for the induction of phase II enzymes through the modulation of ARE sequences [27]. In epithelial bronchial cells (BEAS-2B), the enzymatic metabolite of lycopene apo-10′-lycopenoic acid induces the nuclear accumulation of Nrf2, which associates with phase II detoxifying enzymes and antioxidant enzymes including HO-1, NQO1, GST, and GCL [32]. Moreover, apo-10′-lycopenoic acid increases intracellular levels of GSH, suppresses ROS production, and reduces the oxidative damage induced by H_2_O_2_ in BEAS-2B cells. These data suggest that lycopene-derived metabolites might mediate the activation of Nrf2-ARE signalling and the subsequent induction of gene expression [32]. 

**Figure 1:**
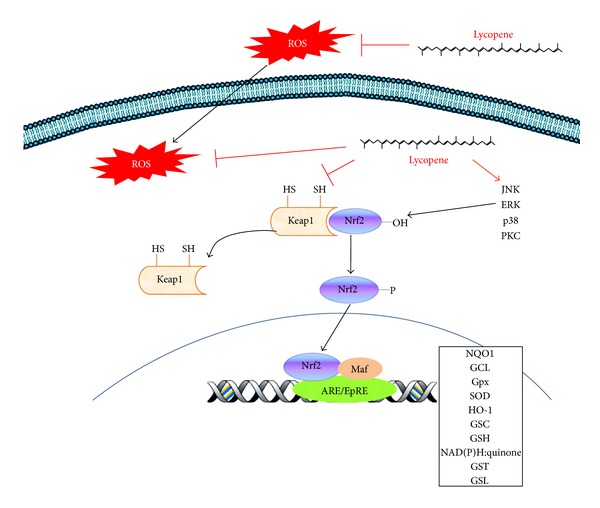
A possible mechanism of Nrf2 regulation by lycopene. Under homeostatic conditions, Nrf2 is retained within the cytoplasm by the Keap1 protein. Upon activation of upstream protein kinases (MAPKs, PI3K, PKC, and ERK) and/or direct effects on Keap1, Nrf2 is released from Keap1 and translocated into the nucleus, where Nrf2 associates with small Maf proteins and binds to AREs. Lycopene induces the nuclear translocation of Nrf2. One possible mechanism involves the direct interaction of lycopene with the cysteine residues of Keap1, which triggers the release of Nrf2 from the complex. Lycopene-generated metabolites can activate a wide variety of kinases, which can also induce the release and nuclear translocation of Nrf2.

Although the molecular mechanism by which lycopene induces the nuclear translocation of Nrf2 is unknown, Lian and Wang [32] have proposed that highly reactive aldehyde groups present in lycopene-derived metabolites form Schiff bases with the N-terminal group of proteins. For example, these reactions may directly modify cysteine residues in Keap1 and thereby abrogate the ubiquitination and subsequent Keap1-mediated degradation of Nrf2. The thiol groups belonging to the seven cysteine residues of Keap1 are oxidised or covalently modified, allowing the dissociation of the Nrf2-Keap1 complex [33]. It is also possible that these lycopenoids affect upstream signalling pathways. For example, lycopene might target MAPKs, PI3K, epidermal growth factor receptor (EGFR), and protein kinase C (PKC), proteins that play an important role in the regulation of Nrf2-ARE signalling in lung epithelial cells [34, 35] (Figure 1).

### 3.2. Growth Factors and Signalling Pathways

Growth factors are proteins, steroids, or any other biochemical substance that might bind to cognate receptors that are present on the cell surface to activate cell signalling cascades that regulate a large variety of cellular processes. Several growth factors, including insulin-like growth factor 1 (IGF-1), vascular endothelial growth factor (VEGF), epidermal growth factor (EGF), and platelet-derived growth factor (PDGF), play important roles in carcinogenesis and metastasis. The abnormal activation of signalling pathways by growth factors leads to increased proliferation, differentiation, maturation, apoptosis suppression, invasion, and metastasis [36].

#### 3.2.1. Effects of Lycopene on IGF Signalling Pathways

Lycopene affects multiple IGF-1-activated signalling pathways. The IGF family of growth factors (IGF-1 and IGF-2) are mitogens that play important roles in the regulation of proliferation, differentiation, and apoptosis. Binding of IGF-1 to its cognate membrane receptor activates the PI3K/AKT/PKB and Ras/Raf/MAP kinase signalling pathways, which in turn regulate several biological processes, including cell-cycle progression, cell survival, and transformation [37]. The IGFs are sequestered in the circulation by a family of IGF-binding proteins (IGFBP-1-IGFBP6) that regulate the binding of IGFs to their receptors [38]. Any alteration of normal physiological IGF-1 signalling leads to increased proliferation and activation of the survival signalling [39]. In breast cancer (MCF-7) and lung cells (NCI-H226), lycopene has been reported to reduce IGF-1 levels and to increase IGFBPs [40, 41]. Lycopene supplementation increases the circulating levels of IGFBP-1 and IGFBP-2 in high-risk populations of colorectal cancer patients, suggesting that lycopene might reduce the risk of colorectal cancer and potentially the risk of other cancers, such as prostate and breast cancer [42]. Moreover, in humans exposed to tobacco smoke, lycopene treatment inhibits lung metaplasia by upregulating IGFBP3 and by decreasing the phosphorylation of BAD, thereby promoting apoptosis and inhibiting cell proliferation. This study suggested that IGFBP3 might inhibit both the PI3K/AKT/PKB and the Ras/Raf/MAP kinase signalling pathways in lung cancer cells, as PI3K mediates the phosphorylation of BAD on serine^136^ and MAPK mediates the phosphorylation of BAD on serine^112^ [43]. Recently, *in vitro* and *in vivo* prostate cancer models have demonstrated that lycopene increases the antineoplastic effects of docetaxel by inhibiting the activation of IGF-1R through the inhibition of IGF-1 stimulation and increased IGFBP3 expression. Furthermore, lycopene might inhibit not only the IGF-1-induced phorylation of IGF-1R but also the downstream activation of AKT and the expression of the antiapoptotic protein survivin in DU145 cells [44].

#### 3.2.2. Effects of Lycopene on PDGF Signalling Pathways

PDGF, another growth factor that is inhibited by lycopenes, is a potent stimulator of the growth and motility of connective tissue cells such as fibroblasts and smooth muscle cells (SMCs). The biologically active form of PDGF is a dimer formed by two polypeptidic chains that are linked by disulfide bonds. It can be either a homodimer (PDGF-AA and PDGF-BB) or a heterodimer (PDGF-AB). This factor exerts its effects on target cells by binding with different specificities to its receptors, PDGFR*α* and PDGFR*β*. Binding of PDGF to PDGFR causes receptor dimerization, autophosphorylation and the activation of receptor tyrosine kinase function, resulting in the subsequent activation of the MAPK and PI3K/AKT- PLC*γ*-PKC signalling pathways [45]. 

Abnormalities in the PDGF-BB-PDGFR*β* signalling pathway contribute to a number of human diseases, including vascular and malignant pathologies [46]. In functional studies, lycopene inhibits PDGF-BB-induced proliferation and migration of SMCs on gelatin and collagen. It also inhibits the phosphorylation of PLC*γ* and ERK1/2 by directly binding to PDGF-BB [47]. In the same way, lycopene inhibits PDGF-BB-induced signalling in cultured human fibroblasts, as well as the phosphorylation of ERK1/2 (kinase regulated by extracellular signals), p38, and JNK (c-jun N-terminal kinase) [48]. Moreover, lycopene inhibits SMC and fibroblast migration by reducing PDGF-AA and PDGF-AB signalling as measured by PDGFR*α* phosphorylation and the activation of downstream kinases [49]. Chiang et al. [50] have shown that lycopene significantly inhibits the fibroblast migration induced by melanoma tumour cells, indicating that lycopene interferes with stroma-tumour cell interactions. Furthermore, lycopene has been suggested to bind to the PDGF secreted by melanoma cells, and it might inhibit PDGF-induced fibroblast migration. Thus, lycopene might help to prevent melanoma progression.

#### 3.2.3. Effects of Lycopene on VEGF Signalling Pathways

VEGF, which is encoded by the VEGF-A, -B, -C, -D, and -E genes, is one of the main regulators of vascular endothelial cells and blood vessel formation, and it is a member of the VEGF-PDGF super family along with the placental growth factor (PLGF). Binding of VEGF to its receptors (VEGFR-1, -2, and -3) results in the activation of the MAPK and PI3K-PLC*γ*-PKC pathways. VEGFR-2 activation increases and activates endothelial nitric oxide synthase (eNOS), which raises the levels of nitric oxide (NO), playing a crucial role in VEGF-induced endothelial cell proliferation, migration, tube formation, vascular permeability increase, hypotension, and angiogenesis [51, 52]. 

Recent studies have shown that lycopene inhibits human umbilical vascular endothelial cell (HUVEC) migration and tube formation [53]. Moreover, high doses of lycopene can inhibit tumour growth in nude mice xenotransplanted with the PC-3 prostate carcinoma and Sk-Hep-1 hepatocellular carcinoma cell lines. In both of these neoplasms, high-dose lycopene treatment also decreases the circulating levels of VEGF. These data suggest that lycopene elicits antiangiogenic effects [54]. Lycopene inhibits angiogenesis by inhibiting matrix metalloproteinase (MMP)-2 and the urokinase plasminogen activator (uPA) system through the inhibition of VEGFR2 signalling pathways, including ERK1/2, p38, and PI3K-AKT and by reducing the expression of Rac-1 protein. These changes reduce the invasion, migration, and tube formation capacity of HUVECs [55]. 

**Figure 2:**
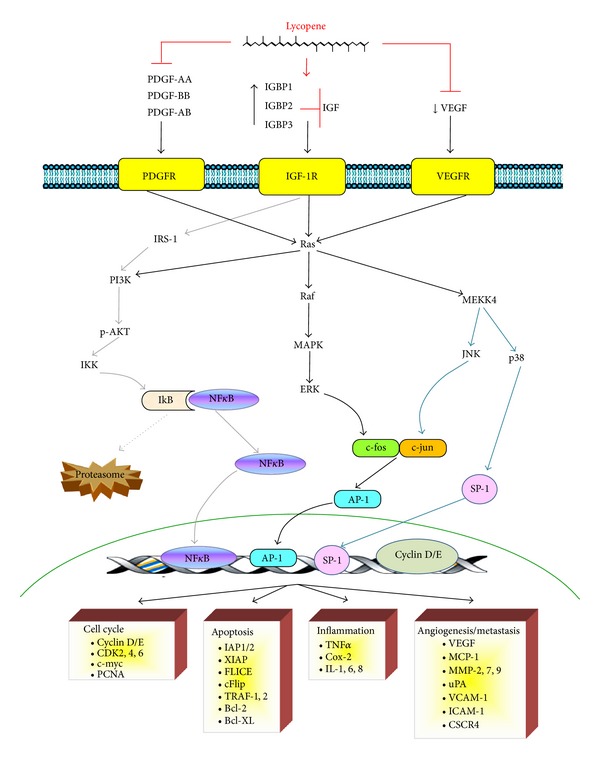
PDGFR-, IGF-IR-, and VEGFR-mediated signal transduction pathways are possible targets for lycopene. PDGFR, IGF-IR, and VEGFR are activated at the cell surface during tumourigenesis. Activation of these receptors induces several downstream signalling pathways. Among these pathways, the Ras-MAPK (including ERK, JNK, and p38) and PI3K-AKT pathways transduce signals into the nucleus to activate transcription factors that regulate the expression of genes that are important for proliferation, cell-cycle progression, apoptosis, inflammation, angiogenesis, invasion, and metastasis. Lycopene has been shown to inhibit the IGF-induced activation of IGFR by increasing the expression of IGFBPs. Similarly, lycopene directly binds to PDGF to reduce the autophosphorylation of PDGFR and VEGFR.

These results suggest that lycopene may inhibit the carcinogenic process through the inactivation of the growth factor (PDGF, VEGF, and IGF) induced PI3K/AKT/PKB and Ras/RAF/MAPK signalling pathways. When active, these pathways activate transcription factors (NF-*κ*kB, AP-1, and SP-1) that regulate the expression of genes that control cellular processes such as proliferation, cell cycle, apoptosis, inflammation, angiogenesis, invasion, and metastasis (Figure 2).

### 3.3. Cell-Cycle Arrest

Cell-cycle progression is regulated through the interaction between cyclin-dependent kinases (CDK1, -2, -3, -4, and -6), cyclins (cyclin A, -B, -D, and -E), and inhibitor proteins (p21^WAF1^ and p27^KIP1^) [56, 57]. The coordinated participation of cyclin D/CDK4/6, cyclin E/CDK2, and cyclin A/CDK2, complexes is required for the G_1_/S transition and progression through S phase, whereas the cyclin A/CDK1/2 complexes are required for cells to enter mitosis. The activation of the cyclin D/CDK4/6 and cyclin E/CDK2 complexes is essential for the phosphorylation of retinoblastoma (Rb). Rb is constitutively expressed and dephosphorylated; it forms a complex with histone deacetylase-1 and the transcription factor E2F, inhibiting its transcriptional activity. By activating ERK1/2, growth factors might induce an increase in the expression of cyclin D, which binds to and activates CDK4 and CDK6. Rb is phosphorylated by these activated CDKs, inducing the dissociation of histone deacetylase-1 from the complex, allowing for histone acetylation and facilitating the transcription of specific genes. These genes include cyclin E, which binds to and activates CDK2. CDK2 then hyperphosphorylates Rb, releasing the E2F transcription factor, which in turn induces the gene expression necessary for the transition to S phase. Among these genes are DNA polymerase A, dihydrofolate reductases, thymidylate synthase, and cyclins [58]. 

The activity of cell cycle kinases is often upregulated in cancer due to the overexpression of cyclins and CDKs or to the inactivation of CDK inhibitors. Specifically, the deregulation and accumulation of proteins involved in the cyclin D-Rb signalling axis are quite common in human cancers, including cancers of the liver, breast, lung, skin, and oesophagus [58].

#### 3.3.1. Effects of Lycopene on Cell Cycle

Lycopene induces cell-cycle arrest. Park and collaborators [59] reported that the growth of human hepatoma Hep3B cells was significantly inhibited by lycopene  treatment, which induced G_0_/G_1_ and S phase arrest. Another study showed that the inhibited cell growth induced by lycopene in MCF-7 cells was associated with a decrease in cyclin D and c-myc expression. In this case, reduced CDK4 activity and retention of p27 within cyclin E-CDK2 complexes resulted in decreased CDK2 kinase activity, leading to reduced Rb phosphorylation and thus inhibition of the G_1_/S transition [60]. Using MCF-7 breast cancer cells and ECC-1 endometrial cells, Nahum et al. [61] found that lycopene and all-trans retinoic acid (ATRA) inhibited IGF-1 stimulated cell-cycle progression through the G_1_ and S phases and also inhibited Rb phosphorylation. These events were associated with a reduction in cyclin D and p21^WAF1^. Moreover, the attenuation of cyclin Dl levels by lycopene and ATRA represents an important mechanism for inhibiting the mitogenic action of IGF-I. Lycopene also interferes indirectly with cell-cycle progression by inhibiting the IGF-1-induced phosphorylation of tyrosine residues within the insulin-1 receptor substrate (IRS-1) and by inhibiting the DNA binding of the AP-1 transcription factor in breast cancer cells [62]. Recent studies have demonstrated that lycopene blocks cells at the G_1_/S phase transition by decreasing cyclin D and increasing p21, p27, and p53 levels in LNCaP prostate carcinoma cells. Lycopene elicits these changes by inactivating Ras via the inhibition of the mevalonate pathway and decreased expression of 3-hydroxy-3-methylglutaryl coenzyme A reductase (HMG-CoA). Lycopene treatment also reduces the farnesylation of Ras, which promotes the cytoplasmic accumulation of Ras and its consequent inactivation. Furthermore, lycopene reduces the Ras-dependent activation of the NF-*κ*B transcription factor, which regulates the transcription of prosurvival genes, including cyclin D, Bcl-2, Bcl-XL, cIAP, and c-myb [63]. In addition, lycopene was shown to inhibit the growth of MDA-MB-231 cells by blocking cell-cycle progression, inhibiting Skp2, and increasing p27 levels [64]. Skp2 is an E3 ligase involved in cell-cycle progression by targeting various cell-cycle regulators, including p27, p21, and FOXO1, for degradation. Skp2 is overexpressed in a variety of human cancers including cancers of the breast, colon, and prostate, as well as lymphoma and melanoma [65]. In A549 nonsmall lung carcinoma cells, lycopene-derived metabolites, such as apo-10′-lycopenic acid, can induce cell-cycle arrest at the G_1_/S, transition. This cell-cycle arrest is associated with a decrease in cyclin E, an increase in the cyclin-dependent kinase inhibitors p21 and p27, and the transcriptional induction of the RAR*β* tumour suppressor gene, which is associated with the increased expression of p21 and p27 [66]. In the androgen-independent prostate cell line DU145, other lycopene-derived products, such as apo-8′-lycopenal and apo-12′-lycopenal, might significantly reduce cell proliferation by altering the cell cycle [67]. 

**Figure 3:**
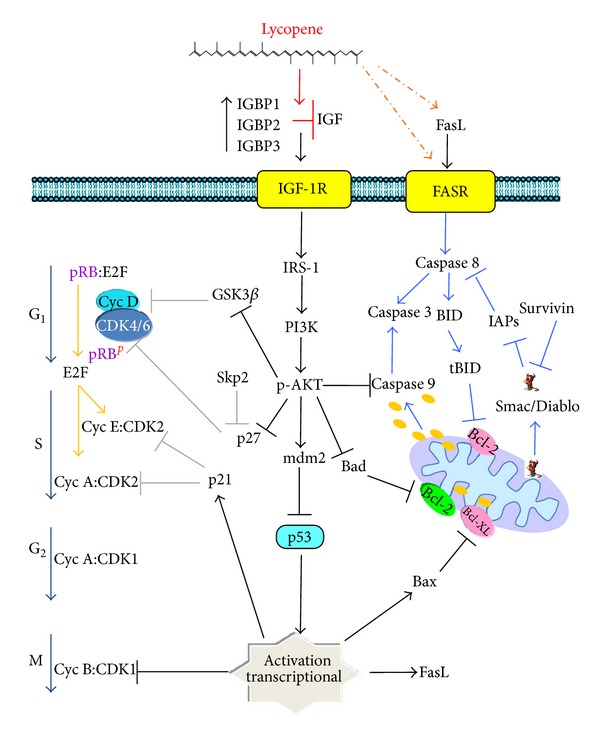
Lycopene induces cell-cycle arrest and apoptosis. Growth factors, such as PDGF and IGF, enhance cell survival by protecting cells from apoptosis. Lycopene blocks cell-cycle progression from G1 to S phase, predominantly by reducing the levels of cyc D and E and subsequently inactivating CDK4 and 2 and reducing the phosphorylation of Rb. Furthermore, lycopene increases the levels of the CDK inhibitors p21 and p27 and the tumour suppressor p53 and reduces levels of SKP2 (leaf panel). Lycopene promotes apoptosis by decreasing Bcl-2, BclXL  and survivin expression, increasing the levels of the proapoptotic proteins Bax, Bad, Bim, and Fas ligand, and activating caspases 8, 9, and 3 (right panel). Lycopene can also block growth factor-mediated antiapoptotic signals by directly inhibiting the binding of growth factors to their receptors or by inhibiting the downstream PI3K-AKT pathway. Lycopene can elicit AKT-induced cell-cycle arrest and apoptosis through the phosphorylation and inactivation of GSK3*β*, p21, p27, Bad, and caspase 9, as well as the inactivation of p53 via Mdm2.

These results suggest that lycopene blocks cell-cycle progression from G_1_ to S phase, predominantly by reducing the levels of cyc D and E and subsequently by inactivating CDK2 and 4 and decreasing the hyperphosphorylation of Rb. Furthermore, lycopene increases the expression of CDK inhibitors including p21 and p27, as well as the tumour suppressor gene p53, and decreases the expression of Skp2 (Figure 3). Lycopene can also block growth-factor-mediated antiapoptotic signals by inhibiting the binding of growth-factors to their receptors or by inhibiting downstream components of the PI3K-AKT pathway. AKT phosphorylates and inactivates glycogen synthase kinase 3*β* (GSK3*β*). Thus by inhibiting GSK3*β*, a growth factor might promote the dephosphorylation and stabilisation of cyc D and c-myc. Cyc D facilitates S-phase entry, and c-myc stimulates cell proliferation and survival. AKT phosphorylates and inactivates the cyclin-dependent kinase inhibitors p21 and p27. An increase in the expression of these inhibitors could in turn inhibit the activity of the CDK 4/6/cyclin D and CDK2/cyclin E complexes and reduce the phosphorylation of Rb. Reduced phosphorylation or hypophosphorylation of Rb leads to the inactivation of the E2F transcription factor and the suppression of the S-phase cyclin A. An additional target of AKT is Mdm2, which mediates the ubiquitination and degradation of p53, which plays a key role in the induction of cell-cycle arrest in response to a variety of genotoxic stresses and to the activation of oncogenes such as c-myc, thereby preventing the propagation of abnormal cells (Figure 3).

### 3.4. Apoptosis

There is a link between the regulation of the cell-cycle and apoptosis: cell cycle deregulation is one of the most potent triggers for apoptosis. Specifically, the deregulation of most components of the cell-cycle machinery, including Rb, E2F, p21, p27, cyclin D, or CDK1, might be involved in the apoptotic process [68]. Apoptosis is regulated by a complex network of pro- and antiapoptotic proteins; it can be induced by either intrinsic or extrinsic pathways. 

Extrinsic pathway is initiated by the interaction of ligands with death receptors such as tumour-necrosis-factor- (TNF-) related apoptosis inducing ligand receptor (TRAILR) and FAS/CD95/APO-1 [69]. All of the death receptors contain intracellular death domains (DDs) that facilitate the transmission of apoptotic signals [70]. The most well-characterised pathway is mediated by the death receptor Fas. Fas ligand (Fas-L) binds to its receptor, inducing the aggregation and activation of other receptors. Once activated, they recruit the adaptor protein FADD, which also contains DDs in addition to a death effector domain (DED), which is required to recruit the initiators of apoptosis, caspases 8 and 10. The assembly of these proteins form the death-induced signalling complex (DISC), which leads to the autoactivation of caspases 8 or 10, which in turn promote the catalytic activation of the effector caspases 3 and/or 7 [71]. Another target of caspase 8 is the proapoptotic protein Bid, which is hydrolysed to tBid inducing Bax oligomerization and depolarization of the mitochondrial with release of cyt c. Together with the activation of caspase 9, these events amplify the apoptotic signal [71]. 

The intrinsic pathway involves the permeabilisation of the mitochondrial external membrane, which facilitates the cytosolic release of proapoptotic proteins such as SMAC/Diablo and cytochrome c (cyt c), which are otherwise confined within the intermembrane space. Cyt c binds to the Apaf-1 protein, which in turn binds to and activates capsase-9, which is responsible for the activation of the executioners of apoptosis, caspases 3, -6, and -7 [68]. 

Both effector pathways of apoptosis are associated with caspase activation. Caspase activation is tightly regulated by inhibitors of apoptosis proteins (IAPs) such as NAIP, cIAP1, cIAP2, XIAP, and survivin. IAPs bind caspases, antagonising their activity. Specifically, XIAP, cIAP1 and cIAP2 inhibit caspase 3, caspase 7, and caspase 9. Survivin inhibits the SMAC/Diablo protein, which binds to and neutralises IAPs [72]. The apoptosis is regulates also by members of the Bcl-2 family that regulate the mitochondrial release of cyt c. Antiapoptotic Bcl-2 family proteins (Bcl-XL and Bcl-2) remain within the external mitochondrial membrane, inhibiting the release of cyt c, whereas proapoptotic Bcl-2 family proteins (Bax, Bim, and Bid) are translocated to the mitochondria to induce apoptosis. Other protein involved in the regulation of apoptosis is the tumour suppressor p53 that transcriptionally activates the proapoptotic genes Bax, Noxa, PUMA, and Bid and transcriptionally represses Bcl-2 and IAPs [73].

#### 3.4.1. Effects of Lycopene on Apoptosis

Lycopene mediates apoptosis via death receptors (Figure 3). Tang et al. [74] reported that lycopene and EPA (eicosapentaenoic acid) synergistically inhibit the activation of AKT and mammalian target of rapamycin (mTOR), enhancing the accumulation of Bax and Fas ligand and blocking the survival of HT-29 human colon cancer cells. Moreover, in combination with S-allyl cysteine, lycopene significantly blocks the *in vivo* development of gastric cancer by inducing apoptosis via reduced expression of Bcl-2, increased expression of Bax and Bim, and increased activation of caspases 8 and 3 [75]. The metabolite (E, E, E)-4-methyl-8-oxo-2, 4, 6-nonatrienal (MON), which is obtained from the autooxidation of lycopene, induces apoptosis in HL-60 human promyelocytic leukaemia cells as evidenced by morphological changes including chromatin condensation and DNA fragmentation, both of which are associated with decreased Bcl-2 and Bcl-XL expression and the activation of caspases 8 and 9. MON was suggested to induce apoptosis via the mitochondrial and death receptor pathways, as it might induce the cytosolic release of cyt c by decreasing the expression of Bcl-2 and Bcl-XL. The enhanced activation of caspase 8 induced by MON might be mediated by death receptors, either by direct binding or by induction of Fas ligand expression [76]. Moreover, lycopene induces apoptosis via the mitochondrial pathway by increasing the expression of tBid and by decreasing the phosphorylation of AKT in canine osteosarcoma cells [77].

A number of studies have shown that lycopene induces apoptotic death via the intrinsic pathway (Figure 3). In the LNCaP cell line, lycopene induces mitochondrial apoptosis in a dose-dependent manner by reducing mitochondrial membrane potential and inducing cyt c release into the cytosol [78]. Recently, combination treatment with lycopene and genistein has been demonstrated to significantly reduce the development of a chemically induced breast cancer *in vivo*. This suppression was associated with decreased Bcl-2 expression, increased Bax expression, and the activation of caspases 3 and 9 [79]. Furthermore, Wang and Zhang [80] reported that lycopene can induce apoptosis in PC-3 cells by altering the cell-cycle distribution, decreasing cyclin D and Bcl-2 expression, and increasing Bax expression. Similarly, Palozza et al. [81] demonstrated that tomato products that contain lycopene can inhibit the growth of colon adenocarcinoma cells by decreasing the expression of cyclin D and the antiapoptotic proteins Bcl-2 and Bcl-XL. They also showed that lycopene induces apoptosis in immortalised fibroblasts by promoting cell-cycle arrest via decreased cyclin D levels and reduced AKT and Bad phosphorylation [82]. Combining lycopene with docetaxel has been shown to induce p53 in LNCaP cells [63] and might synergistically decrease survivin expression levels *in vitro* and *in vivo* [83]. 

These studies have suggested that lycopene inhibits apoptosis by decreasing the expression of Bcl-2, BclXL, and survivin, by increasing the expression of the proapoptotic proteins Bax, Bad, and Bim and Fas ligand and by increasing the activation of caspases 8, 9, and 3. Lycopene can also block growth factor-mediated antiapoptotic signals by directly inhibiting growth factor-receptor binding or by inhibiting downstream components of the PI3K-AKT pathway (Figure 3). AKT can inhibit apoptosis by phosphorylating and inactivating caspase 9 and Bad, a member of the Bcl-2 family of proteins that binds to BclXL and Bcl-2 and inhibits apoptosis by inactivating p53, which regulates the transcription of Bax and FAS ligand (Figure 3). 

### 3.5. Cellular Invasion and Metastasis

Metastasis is the transference of tumour cells from one organ to another. It represents the most severe complication of cancer and is the main cause of death in most cancer patients [84]. Angiogenesis is the initial step in metastasis and is essential for tumour growth and the initial progression of the premalignant tumour towards becoming an invasive cancer. The subsequent steps involve the loss of cellular adhesion in tumour cells, alterations in the basal membrane, infiltration and invasion of the surrounding tissues, and subsequent penetration (intravasation) of the tumour cells into blood or lymphatic vessels, which transport these neoplastic cells into the circulation. The metastatic process proceeds with cell survival in the bloodstream, passage into the capillary terminals of distant organs, and escape from these vessels (extravasation), the colonisation of distal sites and the development of secondary tumours [85]. 

#### 3.5.1. Wnt/*β*-Catenin Signalling in Cancer

Some reports have suggested that the Wnt pathway might lead to cellular deregulation by inducing the expression of several oncogenes. Wnt is a determinant factor in cancer; its activation promotes metastasis [86]. Nuclear *β*-catenin is an indicator of the canonical activity of the Wnt pathway, which participates in the initiation, development, and progression of several tumour types [86]. *β*-catenin is associated with cytoplasmic cadherin domains, and it is linked via *α*-catenin to cell-to-cell junctions within the cytoplasm. *β*-catenin can be released from *α*-cadherin into the cytoplasm, where it integrates into a multiprotein complex containing axin, APC (adenomatous polyposis coli), and GSK3*β*. In a nonstimulated cellular state, GSK3*β* is activated and phosphorylates *β*-catenin, promoting its ubiquitination and proteasomal degradation. Phosphorylation restricts *β*-catenin to the cytoplasm, promoting its degradation and preventing its translocation to the nucleus, and therefore, its action as a transcription factor. The mechanisms described previously maintain low amounts of free *β*-catenin within the cytoplasm [87]. Various stimuli, including Wnt signalling, prevent *β*-catenin degradation. Wnt proteins are modified by lipids and intervene in several development processes through their interaction with the Frizzled and lipoprotein receptor-ligated protein 5 and 6 (LRP-5/6) receptor molecules. The activation of Wnt signalling inactivates GSK3*β* by phosphorylation. Unphosphorylated *β*-catenin accumulates within the cytoplasm and can enter the nucleus, where it can bind to lymphoid enhancer factor/T-cell factor (LEF/TCF) family transcription factors to induce gene expression [88]. Some of the transcriptional targets of *β*-catenin have been implicated in cell proliferation [89] and cellular adhesion [90]. Growth factors also allow for the accumulation of *β*-catenin within the cytoplasm and increase its activity as transcription factor. One effect of the signalling cascades induced by growth factors is the inactivation of GSK3*β*, usually by AKT-mediated phosphorylation [88]. 

#### 3.5.2. Anti-Invasive and Antimetastatic Effects of Lycopene on Colon, Liver, and Prostate Cancers

*In vitro* and *in vivo* studies have suggested promising anti-inflammatory, antiangiogenic, anti-invasive, and antimetastatic effects of lycopene in the development of colon, liver, and prostate cancer. The *in vitro* administration of lycopene reduces inflammatory signalling. Simone et al. [91] reported that lycopene inhibits the mRNA and protein expression of the proinflammatory cytokine IL-8 through the inactivation of the NF-*κ*B transcription factor. Moreover, lycopene treatment was shown to result in reduced ERK1/2, JNK, and p38MAPK phosphorylation and increased expression of PPAR*γ*, resulting in increased PTEN activity and the inactivation of AKT. Lycopene has been suggested to induce NF-*κ*B inactivation by inhibiting the phosphorylation of IKK*α* and IKB*α* and by decreasing the translocation of the NF-*κ*Bp65 subunit from the cytosol to the nucleus. Lycopene also inhibits TNF*α*, cyclooxygenase (COX)-2, inducible nitric oxide synthase (iNOS), and interleukin (IL)-6 secretion [92, 93].

Lycopene inhibits in the progression of colon cancer *in vivo* by decreasing proliferating cell nuclear antigen (PCNA), increasing p21 and the activation of caspase 3, increasing the E-cadherin adhesion molecule, and decreasing nuclear levels of *β*-catenin. Moreover, the effects of lycopene are associated with the suppression of COX-2, prostaglandin E2 (PGE_2_), and ERK1/2 phosphorylation, which is inversely correlated with plasma levels of MMP-9 in tumour-bearing mice [94]. Lycopene also decreases MMP-7 expression in colon cancer cells. The increased MMP-7 expression correlates with malignant progression of human colon cancer. The reduction of MMP-7 expression by lycopene is correlated with reduced stability and increased E-cadherin expression, suggesting that MMP-7 might hydrolyse this adhesion molecule. Furthermore, lycopene decreases MMP-7 and c-myc expression by inhibiting AKT, GSK3*β*, and ERK1/2 phosphorylation with subsequent reduction of the AP-1 and *β*-catenin transcription factors. The effects of lycopene are associated with GSK3*β* activation and *β*-catenin destabilisation; lycopene might induce *β*-catenin degradation through the activation of GSK3*β* [95]. Lycopene might inhibit the growth of colon cancer cells by inactivating the AKT signalling pathway and by increasing the accumulation of cytoplasmic phospho-*β*-catenin; these effects are associated with decreased promoter activity and protein expression of cyclin D1. The authors of this study suggest that lycopene indirectly regulates cytoplasmic *β*-catenin levels by modulating its phosphorylation and subsequent proteasomal degradation [96]. Recent studies indicate that increased levels of *β*-catenin correlate with the downregulation of the Wnt/*β*-catenin pathway due to the proteasomal degradation of *β*-catenin and the subsequent decrease in cyclin D [97], as the cyclin D1 gene might be a target of the *β*-catenin/LEF-1 complex [54]. 

Lycopene inhibits the migration and invasion of SK-Hep-1 hepatoma cells *in vitro*. These effects are associated with the upregulation of the nm23-H1 gene, which is a suppressor of metastasis [98]. Hwang and Lee [97] also demonstrated that lycopene inhibits the adhesion, invasion, and migration of SK-Hep-1 cells. These actions are associated with the decreased activity of MMP-2 and MMP-9. Similarly, Huang et al. [99] demonstrated that lycopene inhibits metastatic tumour growth in the lungs of nude mice that were xenotransplanted with SK-Hep-1 cells. These effects of lycopene were associated with the inhibition of tumour invasion (decreased MMP-9 and upregulated nm23-H1), cell proliferation (decreased PCNA), and angiogenesis (decreased MMP-9 and VEGF, but increased IL-2) in the lungs of nude mice. In SK-Hep-1 cells, apo-8′-lycopene has been shown to exhibit more robust antimetastatic activity than lycopene by inhibiting MMP-2 and MMP-9 expression, increasing the expression of nm23-H1, TIMP-1, and TIMP-2 (MMP tissue inhibitors), suppressing the Monomeric Rho GTPase- and inhibiting the focal adhesion kinase- (FAK-) mediated signalling pathway [100]. Conversely, lycopene has been demonstrated to significantly inhibit the DNA binding of NF-*κ*B and the simulator protein (SP1) and to thereby decrease MMP-9 secretion and reduce the invasive capacity of human hepatoma cells [101]. The authors suggest that these lycopene-induced effects might be attributed to its ability to decrease IGF-1R levels, as the IGF-1 signalling pathway has been shown to activate SP1 and NF-*κ*B, which can in turn increase the expression of MMPs [102]. 

Consistent with this notion, lycopene has been shown to inhibit IGF-1-induced prostate cancer growth by reducing AR (retinoic acid) and *β*-catenin, inhibiting the effects of IGF-1 on the phosphorylation of AKT and GSK3*β* [103] and inhibiting angiogenesis by decreasing VEGF and EGF levels in nude mice that were xenotransplanted with PC-3 cells [104]. 

**Figure 4:**
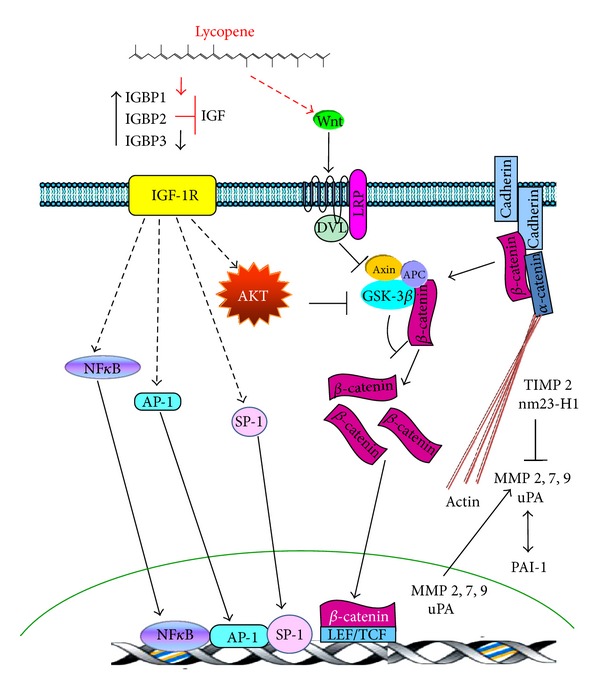
Lycopene inhibits theWnt/*β*-catenin pathway. In the absence of Wnt, a multiprotein complex that includes axin, APC, and GSK3*β* destabilises *β*-catenin. *β*-catenin is phosphorylated by GSK3*β* and is subsequently degraded by the proteasome. Binding of Wnt to its cell surface receptors Fzd and Lrp 5/6 inhibits the phosphorylation of *β*-catenin by GSK3*β*, allowing *β*-catenin to accumulate within the cytosol. *β*-catenin then translocates into the nucleus, where it binds and activates LEF/TCF and induces gene expression. Lycopene increases the expression of E-cadherin, nm23-H1, and TIMP2, as well as the activity of GSK3*β*. Furthermore, it decreases the levels of MMPs 2, 7, and 9, uPA, and *β*-catenin. Lycopene also induces its antimetastatic effects through the inactivation of transcription factors (NF-*κ*B, AP-1, SP-1, and LEF/TCF).

These studies suggest that the regulation of metastatic progression is a target of lycopene for the prevention and therapeutic intervention against cancer (Figure 4). The potential mechanisms underlying the antimetastatic effects of lycopene include the downregulation of the Wnt/*β*-catenin pathway. Lycopene elicits several effects, including the increased expression of E-cadherin, nm23-H1 and tissue inhibitor of metalloproteinases 2 (TIMP2) proteins, and the decreased activity of GSK3*β*, MMP-2, MMP-7, MMP-9, uPA, and *β*-catenin protein (Figure 4). Other potential mechanisms underlying the action of lycopene include blocking the growth factor-mediated activation of the PI3K-AKT pathway and the Akt-mediated phosphorylation of GSK3*β*, which otherwise phosphorylates *β*-catenin to promote its proteasomal degradation and to inhibit its activity as a transcription factor (Figure 4). 

### 3.6. Lycopene as Adjuvant for Cancer Therapy

Antineoplastic therapies, such as radiation and chemotherapy, are treatments given in addition to surgery to suppress tumor relapse through elimination of any remaining cancer cells. These adjuvant therapies are designed to kill actively proliferating cancer cells but are often ineffective for tumor metastasized. Additionally, their effectiveness is often further hampered by associated side effects and the development of treatment resistance. Studies have provided some encouraging data demonstrating that lycopene might revert multidrug resistance (MDR) inducing apoptosis in tumors cells [105] and can either be used alone or combined with additional antineoplastic agents to inhibit tumor invasion and angiogenesis, as well as amelioration of adverse side effects that result from treatment with antineoplastic agents [75, 79, 83].

#### 3.6.1. Synergistic Effects of Lycopene with Natural Anticancer Compounds

Chemoprevention by diet-derived agents that induce apoptosis is a promising strategy to control cancer, due to its potential efficacy and minimal toxicity. 1,2,5-dihydroxyvitamin D_3_ induces HL-60 cell differentiation [106]. However, it was shown that the use of 1,2,5-dihydroxyvitamin D_3_ for the treatment of psoriasis leads to clinical remission; however, this remission is not always durable due to clinical resistance to these agents developed after prolonged treatment, frequently associated with toxic side effects [107]. To overcome these toxic effects, lower doses of the differentiation-inducing agents have been tested. Amir et al. [108] showed that the combination of low concentrations of lycopene with 1,2,5-dihydroxyvitamin D_3_ in HL-60 cells produced a synergistic inhibitory effect on cell proliferation and cell differentiation associated with additive effect on the accumulation of cells in the G_o_/G_1_ phase compared with the same concentration of lycopene or 1,2,5-dihydroxyvitamin D_3_ alone. Tang et al. [109] reported that lycopene and fish oil combined at physiological concentration induced a synergistic inhibitory effect on tumor growth in a mouse xenograft model of colon cancer. The chemopreventive effects of both compounds were associated with increased expression of cell-cycle inhibitors such as p21^CIP1/WAF1^ and p27^Kip1^, as well as suppression of proliferating cell nuclear antigen, *β*-catenin, cyclin D1, and c-Myc proteins. Furthermore, lycopene and fish oil inhibited tumor progression and inflammation through suppression of MMP-7, MMP-9, COX-2, and PGE2. Similar synergistic inhibitory effects on the proliferation of colon cancer cells by lycopene and eicosapentaenoic acid (EPA), a major component in fish oil, even at low concentration were reported by Tang et al. [74]. The inhibitory mechanism was associated with suppression of PI3K/Akt/mTOR signaling pathway and upregulation of apoptotic proteins such as Bax and Fas ligand. Velmurugan et al. [75] demonstrated that the combination of *S*-allylcysteine, an organosulfur constituent of garlic, and lycopene was more effective in suppressing the development of N-methyl-N′-nitro-N′-nitroso-guanidine- (MNNG-) induced gastric cancer through diminution of Bcl-2 expression and increase of Bax, Bim, and caspase 8 expression that either agent alone; furthermore, the induction of apoptosis was achieved at half the dose reported by previous studies [110]. Similarly, the combination of lycopene and genistein, the most bioactive isoflavones of soybeans, decreased 7,12-dimethylbenz(a)anthracene- (DMBA-) induced breast cancer in rats modulating the expression of apoptosis-associated proteins again, and the combination was more effective than the administration of either compound alone. The authors suggested that apoptosis-inducing effect of genistein and lycopene combination may be attributed to the antioxidant and pharmacological properties of the individual agents [79]. 

In addition to these studies, additional reports have analysed the antioxidant or chemopreventive effects of lycopene in combination with other cellular antioxidants. Al-Malki et al. [111] investigated the chemopreventive potential of lycopene and tocopherol on mammary tumorigenesis induced by DMBA in female rats; they found that tocopherol enhanced the ability of lycopene to decrease the levels of both, malondialdehyde, a marker of lipid oxidation, and nitric oxide in serum and breast tissue of DMBA-injected rats. Similar results were reported with combination of lycopene and melatonin; the treatment inhibited lipid-peroxidation and significantly increased the activities of SOD, CAT and GPx [112]. In another study, lycopene and tocopherol co-treatment contribute to the reduction of prostate cancer by interfering with internal autocrine or paracrine loops of sex steroid hormone and growth factor activation and synthesis in the prostate. Furthermore, it has been reported that a cooperative decrease in oxidative stress by the combination of the two antioxidants can explain a significant reduction in JNK signaling [113].

#### 3.6.2. Effects of Lycopene Administration on Chemotherapy

Lycopene sensitizes cancer cells to some chemotherapeutic drugs. Tang et al. [83] showed that lycopene treatment enhanced the growth-inhibitory effect of docetaxel in PCa, *in vitro *in DU145 cells, and *in vivo* in a xenograft model, both models with IGF-IR high expression. Lycopene inhibited IGF-IR activation through inhibition of IGF-I and by increasing the expression and secretion of IGF-BP. Downstream effects include inhibition of AKT kinase activity and survivin expression, followed by apoptosis. Lycopene supplementation also enhances the antitumor activity of cisplatin and ameliorate cisplatin-induced renal oxidative stress in rats [114]. The chemosensitization effect of lycopene has been demonstrated in breast cancer cells. Lycopene increased quinacrine activity and inhibited Wnt-TCF signaling through APC in cancer cells without affecting MCF-10A in normal breast cell line [115]. Similarly, Yang et al. [116] demonstrated that lycopene enhances the antiproliferative effect of LXR*α* agonist T0901317 in DU145 cells and increases the protein expression of PPAR_*γ*_-LXR*α*-ABCA1, leading to high cholesterol efflux. Palozza et al. [117] demonstrated that the inhibition of HMG-CoA reductase by lycopene was also accompanied by a reduction in intracellular cholesterol levels. In addition Yang et al. [118] showed that lycopene increased ABCA1 and apo1 expression and decreased total intracellular cholesterol levels in LNCaP cells suggesting that lycopene is involved in the PPAR-LXR-ABCA1 pathway mediating the cholesterol efflux in LNCaP cells. It has been shown that ABCA1 antitumor activity requires efflux function and appears to be mediated by reduction of mitochondrial cholesterol combined with the increased release from mitochondria of cell death promoting molecules, such as cyt c [119]. 

#### 3.6.3. Effects of Lycopene Administration on Radiotherapy

Radiation therapy is one of the most widely used anticancer therapies; it is often associated with acute side effects that may decrease tolerance to the treatment, reduction of the optimal dose and volumen of radiation, or interruption of planned treatment. Andic et al. [120] demonstrated that lycopene may decrease the severity of radiation-induced acute toxicity in the gastrointestinal tract, weight loss, and diarrhea in rats. Interestingly, pretreatment with lycopene protects the lymphocytes from *γ*-radiation-induced damage by inhibiting the peroxidation of membrane lipids, the accumulation of free radicals, and DNA strand break formation [121]. Likewise, lycopene has shown protective effects against ultraviolet radiation [122].

Intriguingly, lycopene appears to protect from side effects, whereas at the same time sensitizes the tumor cells towards radiation-induced cell death. Camacho-Alonso et al. [123] demonstrated that lycopene increases cytotoxic activity of radiotherapy in oral squamous cell carcinoma cells and reduces its invasiveness [123]. Similarly, Tabassum et al. [124] reported that lycopene exerts a synergic effect when combined with radiation, in certain types of tumors such as HNSCC and prostate cancer. These studies suggest that lycopene might participate simultaneously as a radioprotector for normal cells and radiosensitizer for cancer cells. 

## 4. Conclusion

**Figure 5:**
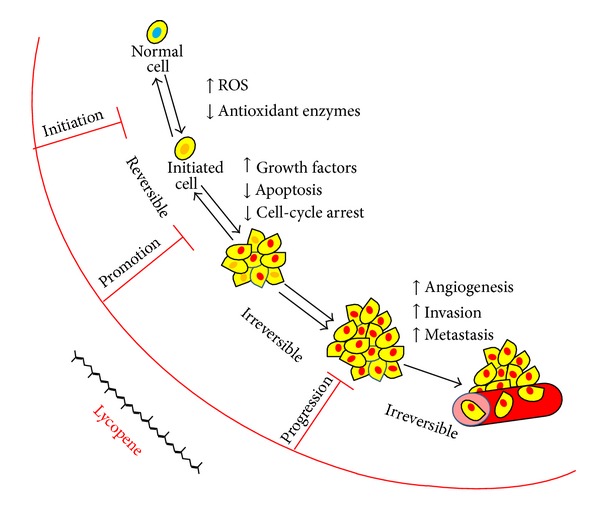
Phases of lycopene intervention in the carcinogenic process. Cancer development is a multistep process that includes initiation, promotion, and progression. The initiation step is started by the addition of a carcinogen or irradiation in normal cells. Lycopene and its metabolites can block this step by inactivating ROS and inducing the detoxification and antioxidant enzyme systems that protect cells from the damage caused by carcinogenic initiators. Lycopene can also block or impede tumour promotion and progression by modulating key signalling pathways induced by tumour promoters, inflammatory cytokines, and growth factors.

The gradual increase in understanding of cancer biology during recent years has resulted in the elucidation of several approaches for intervention in carcinogenesis. The antioxidant, anti-inflammatory, and proapoptotic activities of lycopene and its metabolites might contribute to the prevention of and therapy for cancer by modulating diverse biochemical processes involved in carcinogenesis (Figure 5). The potentially beneficial effects of lycopene include the inhibition of carcinogenic activation, proliferation, angiogenesis, invasion and metastasis, the blocking of tumour cell-cycle progression, and the induction of apoptosis through alterations in various signalling pathways.

More large-scale clinical trials are required to fully evaluate the potential value of lycopene and its metabolites in the prevention and treatment of cancer, to determine the optimal dosing and route of administration and to identify cancer targets and potential interactions with other drugs.
